# Expiratory flow limitation in intensive care: prevalence and risk factors

**DOI:** 10.1186/s13054-019-2682-4

**Published:** 2019-12-05

**Authors:** Carlo Alberto Volta, Francesca Dalla Corte, Riccardo Ragazzi, Elisabetta Marangoni, Alberto Fogagnolo, Gaetano Scaramuzzo, Domenico Luca Grieco, Valentina Alvisi, Chiara Rizzuto, Savino Spadaro

**Affiliations:** 1 Department of Morphology, Surgery and Experimental Medicine, Azienda Ospedaliera-Universitaria Arcispedale Sant’Anna, University of Ferrara, Via Aldo Moro, 8, 44124 Ferrara, Italy; 2 Department of Anesthesiology and Intensive Care Medicine, Catholic University of The Sacred Heart, Milan, Italy; 3 Department of Anesthesia and Intensive Care Unit, ASST Fatebenefratelli Sacco, Luigi Sacco Hospital, Polo Universitario, University of Milan, Milan, Italy

**Keywords:** Respiratory insufficiency, Fluid therapy, Lung disease, Respiratory mechanics, Maximal expiratory flow rates, Critical care

## Abstract

**Background:**

Expiratory flow limitation (EFL) is characterised by a markedly reduced expiratory flow insensitive to the expiratory driving pressure. The presence of EFL can influence the respiratory and cardiovascular function and damage the small airways; its occurrence has been demonstrated in different diseases, such as COPD, asthma, obesity, cardiac failure, ARDS, and cystic fibrosis. Our aim was to evaluate the prevalence of EFL in patients requiring mechanical ventilation for acute respiratory failure and to determine the main clinical characteristics, the risk factors and clinical outcome associated with the presence of EFL.

**Methods:**

Patients admitted to the intensive care unit (ICU) with an expected length of mechanical ventilation of 72 h were enrolled in this prospective, observational study. Patients were evaluated, within 24 h from ICU admission and for at least 72 h, in terms of respiratory mechanics, presence of EFL through the PEEP test, daily fluid balance and followed for outcome measurements.

**Results:**

Among the 121 patients enrolled, 37 (31%) exhibited EFL upon admission. Flow-limited patients had higher BMI, history of pulmonary or heart disease, worse respiratory dyspnoea score, higher intrinsic positive end-expiratory pressure, flow and additional resistance. Over the course of the initial 72 h of mechanical ventilation, additional 21 patients (17%) developed EFL. New onset EFL was associated with a more positive cumulative fluid balance at day 3 (103.3 ml/kg) compared to that of patients without EFL (65.8 ml/kg). Flow-limited patients had longer duration of mechanical ventilation, longer ICU length of stay and higher in-ICU mortality.

**Conclusions:**

EFL is common among ICU patients and correlates with adverse outcomes. The major determinant for developing EFL in patients during the first 3 days of their ICU stay is a positive fluid balance. Further studies are needed to assess if a restrictive fluid therapy might be associated with a lower incidence of EFL.

## Background

Expiratory flow limitation (EFL) is a dynamic condition in which expiratory flow has already reached its maximal value [1]. According to Mead et al. [2], once the expiratory flow is limited at a given lung volume, there is a site in the intrathoracic airways where intrabronchial and extrabronchial pressure are equal, the so-called equal pressure point (EPP) [3]. Airways downstream of the EPP would be compressed, the diameter markedly reduced, with the expiratory flow becoming insensitive to increases of expiratory driving pressure or to the contraction of the expiratory muscles [1, 4–7].

Clinically, EFL was demonstrated in patients with chronic obstructive pulmonary disease (COPD) [4, 8], acute respiratory distress syndrome (ARDS) [9, 10], acute and chronic heart failure [11, 12], cystic fibrosis [13], spinal cord injury [14] and obesity [15]. Recently, EFL has been described in patients undergoing general anaesthesia for major abdominal surgery, and its presence was the best predictors of postoperative pulmonary complications [16].

The mechanisms leading to EFL can vary among different pathologies. COPD patients may develop EFL because of increased expiratory resistance [17] that tend to reduce the transmural pressure (i.e. the difference between the pressure inside and outside the airways), leading to the development of the EPP. Incomplete lung emptying is frequently associated with dynamic lung hyperinflation with the generation of intrinsic positive end-expiratory pressure (PEEPi) [18]. The latter can have several adverse effects on haemodynamic (i.e. cardiac output depression, increased pulmonary vessel resistance), respiratory muscle function (i.e. altered length-tension characteristics of the diaphragm, increased work of breathing) and patient-ventilator interaction (i.e. patient-ventilator asynchrony). On the other hand, patients with ARDS [19] or those undergoing general anaesthesia [20] can experience a reduction of functional residual capacity (FRC) able both to increase the expiratory resistance and to favour the collapse of the small airways. The ensuing inspiration re-open those airways, and repetitive opening and closure of small airways has been shown to induce histological damage of small airways probably due to the development of high shear forces [21, 22]. This should elicit an inflammatory response and increase the risk of low lung volume injury [23, 24].

Although EFL seems to represent a relevant pathological condition, surprisingly, only few studies evaluated the prevalence of EFL in critically ill patients. Alvisi et al. [8] demonstrated that almost every COPD patient (93%) is flow limited at intensive care unit (ICU) admission for an acute and chronic respiratory failure, while Koutsoukou et al. [9] found that EFL might be common in patients with ARDS. However, both studies enrolled a small number of selected patients so that it is difficult to derive conclusions on the clinical relevance of EFL and its determinants.

The primary aim of the present study is to evaluate the prevalence of EFL in ICU patients requiring mechanical ventilation for acute respiratory failure, and to determine the main clinical characteristics and risk factors associated with the presence of EFL. Secondly, we explored the possible impact of the presence of EFL on patients’ clinical outcome.

## Methods

### Design, setting and patients

We performed a prospective, observational study conducted in the general ICU of the S. Anna University Hospital, Ferrara, Italy. The study was approved by the ethics committee of our institution (Azienda Ospedaliero-Universitaria Ferrara Ethic Committee, protocol number: 74/2016). Informed consent was obtained from each patient or next of kin. Patients were recruited over a 12-month period between April 2016 and April 2017.

We screened and included all consecutive patients admitted to the ICU older than 18 years with an acute respiratory failure and with an expected length of mechanical ventilation of 72 h or more, as judged by the physician in charge. Exclusion criteria were (1) pregnancy, (2) haemodynamic instability (i.e. heart rate ≥ 120 beats/min or cardiac arrhythmia; systolic blood pressure < 90 or vasopressor use, i.e. dopamine or dobutamine ≥ 5 μg/kg/min or noradrenaline ≥ 0.1 μg/kg/min), (3) presence of laparostomy and (4) active air leakage (i.e. pneumothorax or presence of thoracic drainage) (Additional file 1).

The observational period started within 24 h from admission to ICU and continued for at least 72 h. Patients were followed for outcome assessment until hospital discharge.

### Determination of EFL and respiratory variables

All measurements were performed by three investigators (FDC, CR, EM) equally expert in respiratory mechanics and data collection. Patients were studied in a semi-recumbent position, with a head of bed angle of 30°.

**Fig. 1:**
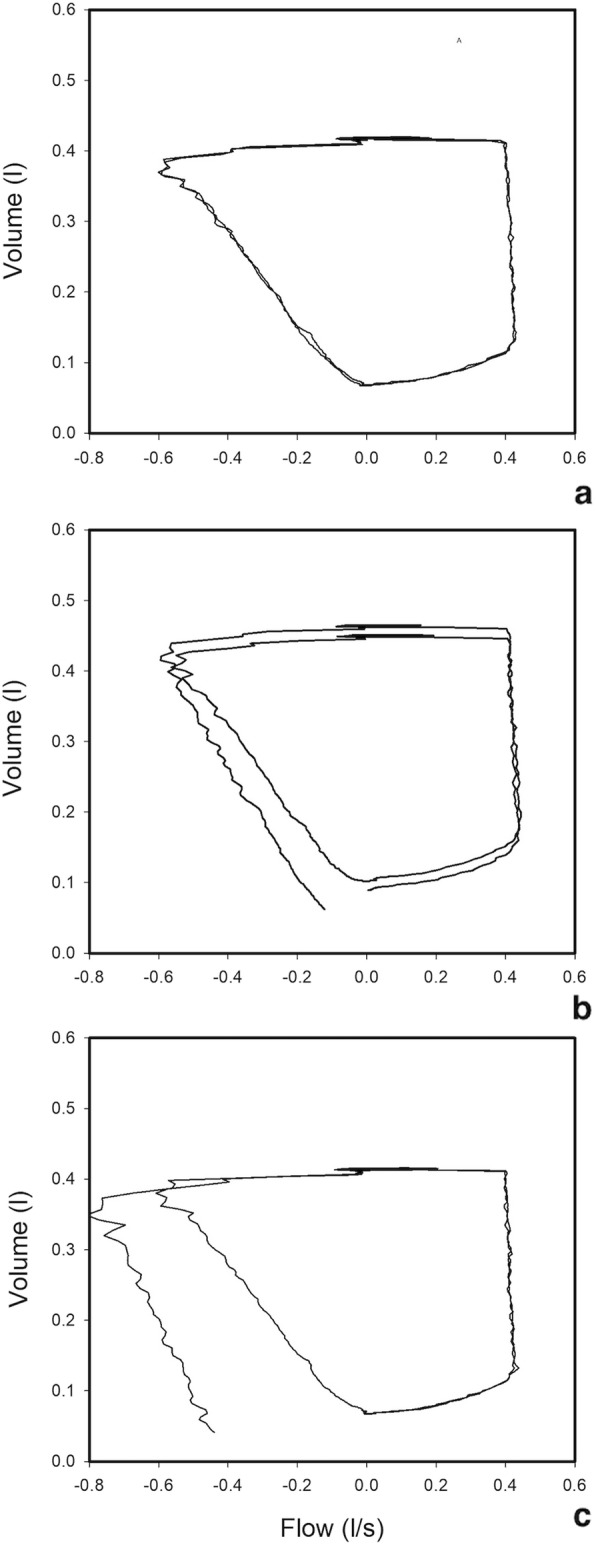
Flow-volume curves of a representative patient aimed to determine the level of PEEP able to eliminate the presence of EFL (PEEP-EFL). **a** Until the subtraction of 4 cmH_2_O of PEEP the expiratory flow did not increased: the patient was considered flow limited at 4 cmH_2_O of PEEP. **b** Subtraction of 6 cmH_2_O of PEEP increased the expiratory flow. **c** Subtraction of 8 cmH_2_O increased the expiratory flow more than after the subtraction of 6 cmH_2_O of PEEP. The PEEP-EFL was 5 cmH_2_O. See text for further explanation

The presence of EFL was determined by the PEEP test. The latter is based on a sudden decrease of PEEP from 3 to 0 cmH_2_O at the end of inspiration in order to increase the expiratory driving pressure and establish whether or not the expiratory flow increases. If the expiratory flow increases after subtraction of PEEP, then the patient is classified as not flow limited. On the contrary, if the expiratory flow does not increase after subtraction of PEEP, the patient is classified as having EFL. This approach requires a specific manoeuvre to show two different flow-volume loops in the same display, and it is available on all modern ventilators. The flow-volume curve with 3 cmH_2_O of PEEP is used as a reference and fixed on the screen. The flow-volume curve of the ensuing breath in which PEEP is reduced to 0 cmH_2_O is superimposed to the previous one in order to determine if the two flow-volume curves overlap (i.e. the expiratory flow does not increase), or the flow-volume curve at ZEEP exhibit an increase of the expiratory flow (patient not flow limited) (Additional file 2). The accuracy and the reproducibility of the PEEP test have been compared to the Negative Expiratory Pressure (NEP) test and validated previously [20]. Further, the same PEEP test was used to determine the value of PEEP able to eliminate the presence of EFL, the so-called PEEP-EFL. The latter was calculated as the minimal value of PEEP that, according to the flow-volume curve, allows the expiratory flow to increase during tidal expiration (Fig. 1). This was obtained by an incremental PEEP trial.

Respiratory mechanics were performed at zero-PEEP (ZEEP) by the standard airway occlusion technique using a 5-s end-expiratory occlusion followed by a 5-s end-inspiratory occlusion [20]. The flow and additional resistance as well as the static compliance of the respiratory system were computed using standard formulas [20]. During these tests, patients were deeply sedated using continuous intravenous infusion of propofol (1–2 mg/kg) and paralysed with a bolus of rocuronium bromide (0.6 mg/kg) and mechanically ventilated in volume-controlled mode. Patients with COPD were studied after at least 8 h from the administration of albuterol. As per our clinical practice, patients without COPD were not given bronchodilators.

The severity of chronic dyspnoea was rated according to the modified dyspnoea scale proposed by the Medical Research Council (mMRC) [25].

### Data collection and outcome data

The presence of EFL was determined at the ICU admission (within 12 h) and daily during the first 72 h. Data of respiratory mechanics were assessed at day 1 and at day 3 from ICU admission.

Demographics, anthropometrics, comorbidities, information and causes of hospitalisation were recorded into study-specific case report forms and database. COPD was defined according to recent ATS/ERS criteria [26], and COPD severity was assessed by the Global Initiative for COPD (GOLD) criteria [27]. Simplified Acute Physiology Score (SAPS) II and Sequential Organ Failure Assessment (SOFA) were determined during the first 24 h after ICU admission. The diagnosis of ARDS was based on the Berlin definition [28]. The occurrences of acute kidney injury (AKI) and septic shock were diagnosed according to international guidelines statements, Kidney Disease: Improving Global Outcomes (KDIGO) criteria [29] and surviving sepsis campaign (Sepsi-3) criteria [30], respectively.

Daily fluid balance was recorded as the algebraic sum of fluid intake and output per day, not including insensible losses, while cumulative fluid balance (CFB) was calculated as the algebraic sum of daily fluid balance during the observational period. We reported CFB as absolute number or divided by the admission weight of the patient (CFB/kg). Cumulative fluid overload (CFO) was calculated by dividing the CFB by the admission weight of each patient and was expressed as a percentage, as previously proposed [31]. We considered a CFO ≥ 10% as severe fluid overload.

Outcome data such as days of mechanical ventilation, ICU and hospital length of stay and ICU and hospital mortality were retrieved from the hospital’s electronic patient chart.

### Statistical analysis

Data are presented as frequencies and percentages and mean ± standard deviation or medians with 25th to 75th percentiles range [interquartile range], depending on the type of data and their distribution. The Shapiro-Wilk test was used to assess the assumption of normality. Categorical data were compared using the *χ*^2^ test or Fisher exact test as appropriate. Unpaired Student’s *t* tests or Mann-Whitney *U* tests for data with normal or non-normal distribution, respectively, were used to compare continuous variables.

Friedman test was used to test differences in CFB, CFB/kg and CFO within groups among three different time points (24 h, 48 h and 72 h from ICU admission). Kruskal-Wallis test and Mann-Whitney test were used to test differences in CFB, CFB/kg and CFO between groups at three different time points (24 h, 48 h and 72 h from ICU admission). Correlation between CFB/kg and intrinsic PEEP was assessed by linear regression.

The association between the presence of EFL at admission and baseline patient characteristics was modelled using binary logistic regression analysis and reported as estimated odds ratio (OR) and relative 95% confidence interval (CI). Patients’ characteristics independently associated with the presence of EFL at ICU admission were assessed in a multivariate logistic regression model. In the same fashion, a univariate logistic approach was used to assess the association between a CFO ≥ 10%, the development of AKI in ICU, AHRF, ARDS or septic shock at admission and the incidence of EFL during the first 72 h of ICU stay.

Statistical analyses were performed using SPSS 20.0 statistical software (SPSS Inc., Chicago, IL). In all statistical analyses, a 2-tailed test was performed and the *p* value ≤ .05 was considered statistically significant.

## Results

A total of 121 patients were enrolled, and their main characteristics at admission are shown in Table 1. The most frequent causes for ICU admission were acute hypoxaemic respiratory failure (AHRF) (43%), sepsis (37%), ARDS (24%) and haemorrhagic shock (9%). Among the 121 patients included, 28 had a diagnosis of COPD and 6 of them were admitted for an acute exacerbation of COPD.

**Table 1:** Clinical and demographic characteristics of the patients at ICU admission

Variables	Total (*n* = 121)	NO EFL (*n* = 84)	EFL (*n* = 37)	*p value*
Age	68 ± 14	67 ± 15	71 ± 12	0.181
Male sex, *n* (%)	81 (67)	60 (71)	21 (57)	0.114
BMI, kg/m^2^	27.0 ± 5.6	25.3 ± 3.9	30.7 ± 6.8	< 0.0001
SOFA at admission	6 [4–9]	6 [4–8]	8 [6–10]	0.015
SAPSII	42 [31–48]	38 [29–47]	42 [35–53]	0.077
Smoking history, *n* (%)				0.343
Current smoker	29 (24)	17 (20)	12 (32)	
Former smoker	31 (26)	16 (25)	15 (26)	
mMRC *≥ 3*	35 (29)	9 (11)	26 (70)	< 0.0001
NYHA *≥ 2*	62 (51)	27 (32)	35 (95)	< 0.0001
Comorbidities, *n* (%)				
Heart diseases	68 (56)	40 (48)	28 (76)	0.004
Hypertension	42 (35)	28 (33)	14 (38)	0.632
Chronic cardiac ischaemia	43 (36)	22 (26)	21 (57)	0.001
COPD	28 (23)	9 (11)	19 (51)	< 0.0001
OSAS	7 (6)	3 (4)	4 (11)	0.116
CKD	21 (17)	10 (12)	11 (30)	0.017
Reason for MV initiation, *n* (%)				
AHRF	52 (43)	31 (37)	21 (57)	0.042
Sepsis	45 (37)	34 (41)	11 (30)	0.260
Septic shock	28 (23)	21 (25)	7 (19)	0.465
Haemorrhagic shock	11 (9)	8 (10)	3 (8)	0.803
Coma	13 (11)	11 (13)	2 (5)	0.208
ARDS	29 (24)	13 (16)	16 (43)	0.001
Mild	7 (24)	3 (23)	4 (24)	
Moderate	14 (48)	8 (62)	6 (38)	
Severe	8 (28)	2 (15)	6 (38)	

*EFL* expiratory flow limitation, *BMI* body mass index, *SOFA* Sequential Organ Failure Assessment, *SAPS II* Simplified Acute Physiology Score, *mMRC* modified Medical Research Council dyspnoea scale, *NYHA* New York Heart Association classification, *COPD* chronic obstructive pulmonary disease, *OSAS* obstructive sleep apnoea syndrome, *CKD* chronic kidney disease, *ICU* intensive care unit, *AHRF* acute hypoxaemic respiratory failure, *ARDS* acute respiratory distress syndrome

### Occurrence of EFL

Upon admission, 37/121 (31%) patients exhibited EFL, with a median PEEP-EFL of 8 cmH_2_O [6–10]. Among the patients having EFL at admission, 19/37 (51%) had a diagnosis of COPD. Compared to those without EFL, flow-limited patients had a higher body mass index (BMI) (30.7 ± 6.8 vs 25.3 ± 3.9, *p* < 0.0001) and worse respiratory dyspnoea score [mMRC ≥ 3 26/37 (70%) vs 9/84 (11%), *p* < 0.0001]. EFL was more frequently associated with history of heart disease (28/37 (76%) vs 40/84 (48%), *p* = 0.004), COPD (19/37 (51%) vs 9/84 (11%), *p* < 0.0001) and chronic kidney disease (11/37 (30%) vs 10/84 (12%), *p* = 0.017). Furthermore, the main factors independently related to EFL at ICU admission were a BMI ≥ 30 kg/m^2^ (OR 3.6, 95% CI 1.0–12.6, *p* = 0.049), a mMRC score ≥ 3 (OR 8.0, 95% CI 2.3–27.1, *p* = 0.001), a SOFA score ≥ 6 (OR 3.6, 95% CI 1.1–12.0, *p* = 0.036) and a medical history of COPD (OR 4.7, 95% CI 1.5–14.4, *p* = 0.008) (Table 2).

**Table 2:** Association between baseline characteristics of patients and the presence of EFL at ICU admission according to logistic regression analysis adjusted for potential confounders

Variables	Univariate analysis			Multivariate analysis		
	Crude odds ratio	95% CI	*p* value	Adjusted odds ratio	95% CI	*p* value
BMI *(ref: < 30 kg/m*^*2*^*)*						
≥ 30 kg/m^2^	7.0	2.8–17.3	< 0.0001	3.6	1.0–12.6	0.049
mMRC *(ref: < 3)*						
≥ 3	19.7	7.3–52.8	< 0.0001	8.0	2.3–27.1	0.001
COPD *(ref: absence)*						
Presence	8.8	3.4–22.6	< 0.0001	4.6	1.4–15.3	0.008
Heart disease *(ref: absence)*						
Presence	3.7	1.6–8.3	0.002	1.6	0.5–5.0	0.418
CKD *(ref: absence)*						
Presence	3.1	1.2–8.2	0.021	1.7	0.4–6.7	0.470
SOFA score *(ref: < 7)*						
≥ 6	3.0	1.2–7.3	0.016	3.6	1.1–12.0	0.036
OSAS *(ref: absence)*						
Presence	3.3	0.7–15.4	0.134			
Age *(ref: < 70)*						
≥ 70	0.9	0.5–2.1	0.968			
Smoking history *(ref: non-smoker)*						
Actual smoker	1.8	0.7–4.6	0.203			
Past smoker	0.9	0.3–2.4	0.834			
SAPS II *(ref: < 41)*						
≥ 42	1.7	0.7–3.7	0.188			

*BMI* body mass index, *mMRC* modified Medical Research Council scale for dyspnoea, *COPD* chronic obstructive pulmonary disease, *CKD* chronic kidney disease, *OSAS* obstructive sleep apnoea syndrome, *SOFA* Sequential Organ Failure Assessment, *SAPS* Simplified Acute Physiology Score

During the first 72 h of ICU stay, 21 additional patients (17%) developed EFL. Specifically, 13 patients (11%) became flow limited after 48 h and 8 (7%) after 72 h. Their clinical characteristics are reported in Additional file 2. Of note, they exhibited a BMI similar to the one of patients without EFL and the diagnosis of COPD (Additional file 3). Finally, patients becoming flow limited during the ICU stay exhibited values of PEEP-EFL statistically lower than those with EFL at ICU admission (Additional file 4).

### Data of respiratory mechanics

At the ICU admission, patients with EFL exhibited higher PEEPi (7 [4–10] vs 2 [1–2] cmH_2_O, *p* < 0.0001), total airways resistance (22 [17–26] vs 16 [13–21] cmH_2_O/l/s *p* < 0.0001) and additional resistance (10 [6–14] vs 7 [5–10] cmH_2_O/l/s, *p* = 0.001) (Table 3). These differences remained constant during the ICU stay, being detected also at 72 h after ICU admission (Table 3). Of note, data of respiratory mechanics of patients developing EFL during the ICU stay were not different from those obtained in the absence of EFL (Additional file 5).

**Table 3:** Data of respiratory mechanics at day 1 and day 3 after ICU admission

Variables	Day 1			Day 3		
	NO EFL (*n* = 84)	EFL (*n* = 37)	*p* value	NO EFL (*n* = 63)	EFL (*n* = 58)	*p* value
Cst,rs, ml/cmH_2_O	49 [40–64]	47 [38–56]	0.309	48 [39–61]	52 [39–56]	0.652
Rrs,max, cmH_2_O/l/s	16 [13–21]	22 [17–26]	< 0.0001	17 [15–20]	21 [17–27]	0.031
Rrs,min, cmH_2_O/l/s	8 [6–12]	9 [7–13]	0.269	9 [7–13]	9 [7–14]	0.825
ΔRrs, cmH_2_O/l/s	7 [5–10]	10 [6–14]	0.001	7 [5–10]	11 [6–14]	0.008
P/F ratio	257 [177–370]	168 [123–260]	0.003	230 [170–329]	183 [134–265]	< 0.0001
PEEP_i_, cmH_2_O	2 [1–2]	7 [4–10]	< 0.0001	1 [0–2]	6 [4–9]	< 0.0001
PEEPappl, cmH_2_O	7 [6–8]	10 [8–12]	< 0.0001	8 [6–10]	10 [8–12]	< 0.0001
RR, breaths/min	15 [14–18]	15 [14–16]	0.839	15 [14–17]	16 [15–20]	0.065
V_T_, ml/kg IBW	7.3 [6.7–7.3]	8.0 [7.0–8.9]	0.098	7.2 [6.4–8.0]	7.3 [6.1–7.9]	0.673
Ppeak, cmH_2_O	20 [17–24]	27 [24–31]	< 0.0001	21 [17–25]	29 [22–33]	< 0.0001
Pplat, cmH_2_O	17 [15–19]	19 [16–18]	0.009	17 [15–20]	19 [15–21]	0.227
Δ*P*, cmH_2_O	9 [8–12]	11 [8–13]	0.268	9 [8–12]	9 [7–12]	0.741

*EFL* expiratory flow limitation, *Cst,rs* static compliance of the respiratory system, *Rrs,max* total resistance of the respiratory system, *Rrs,min* flow resistance of the respiratory system, *ΔRrs* additional resistance of the respiratory system, *P/F* arterial partial oxygen pressure to fraction of inspired oxygen ratio, *PEEPi* intrinsic positive end-expiratory pressure, *PEEP appl* positive end-expiratory pressure applied at the ventilator, *RR* respiratory rate, *V*_*T*_ tidal volume, *IBW* ideal body weight, *Ppeak* peak inspiratory pressure, *Pplat* plateau pressure, ***Δ****P* driving pressure

### Cumulative fluid balance and EFL development

**Fig. 2:**
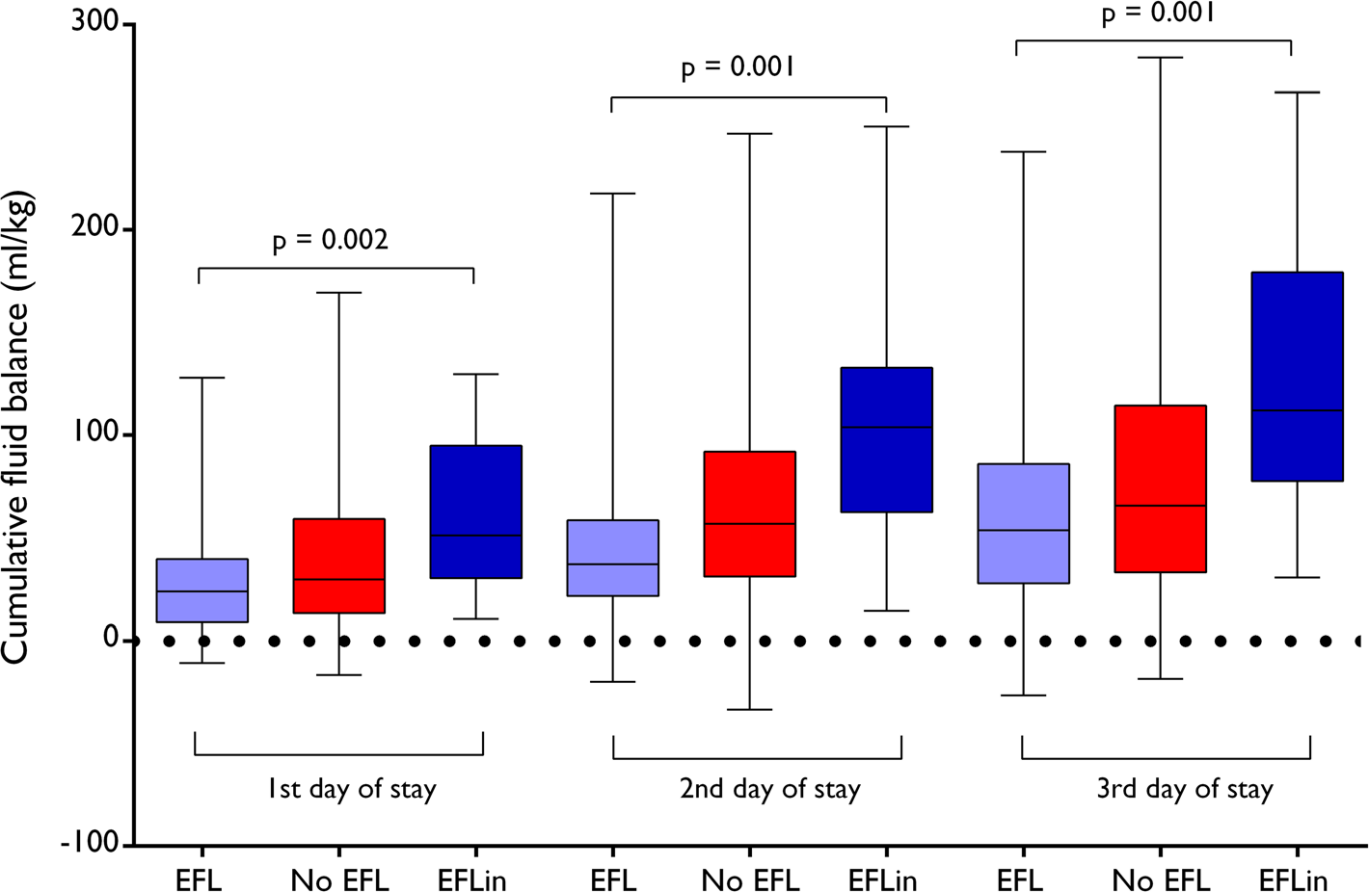
Cumulative fluid balance over the first 3 days of ICU stay. Patients who developed expiratory flow limitation (EFL) after ICU admission (blue) had higher cumulative fluid balances compared to those flow limited at admission (violet), and those who never developed EFL (red)

Patients who developed EFL during the first 72 h of ICU stay had a higher cumulative fluid balance and cumulative fluid overload compared to patients without EFL and with EFL at ICU admission. The trend of cumulative fluid accumulation is shown in Fig. 2 and in Additional file 6.

**Fig. 3:**
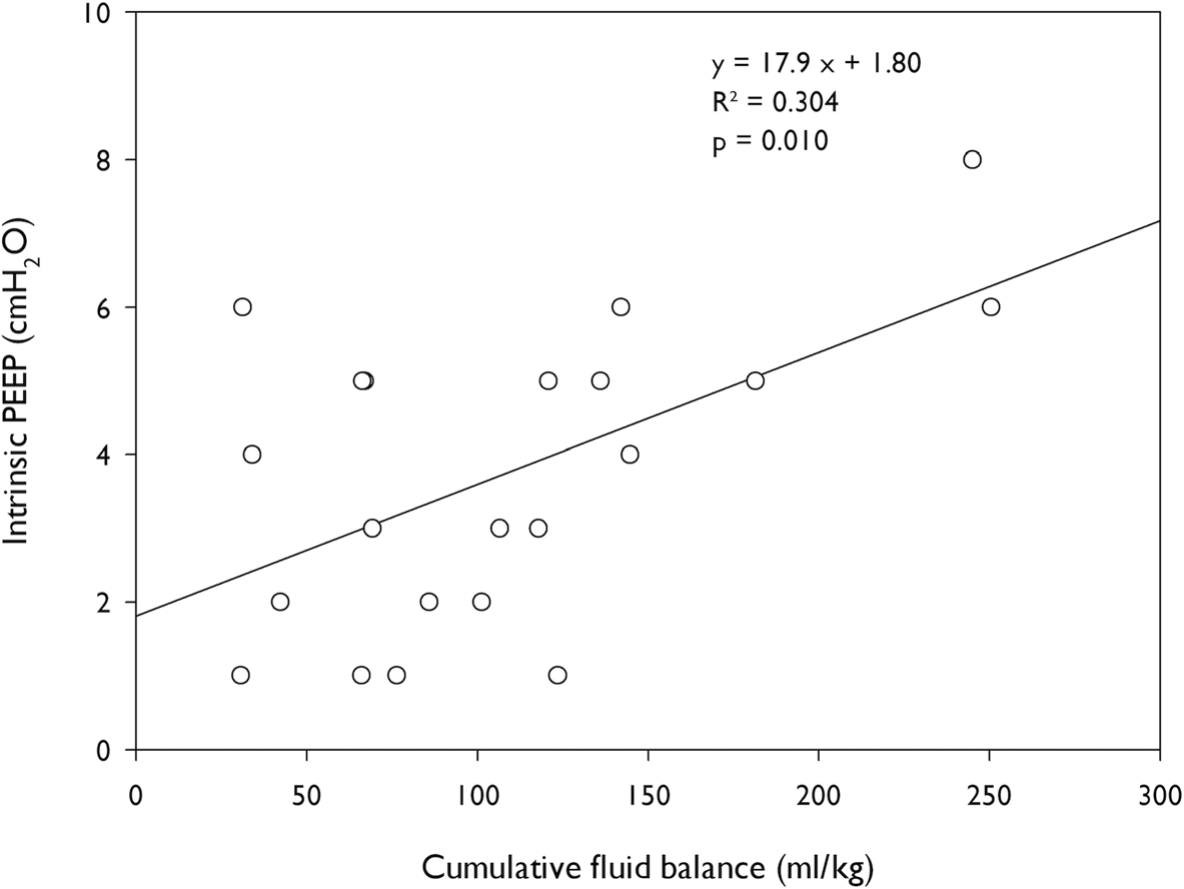
Correlation between CFB and values of PEEPi in patients developing EFL after the ICU admission. The correlation was determined the day the patients became flow limited

In patients developing EFL during the ICU stay, a higher cumulative fluid balance was associated with higher values of intrinsic PEEP on the day of EFL development (*R*^2^ = 0.304, *p* = 0.010) (Fig. 3).

Moreover, a CFO ≥ 10% over the first 2 and 3 days of ICU stay was associated with the development of EFL over the first 3 days of stay in ICU (OR 3.9, 95% CI 1.4–10.9, *p* = 0.011 and OR 3.1 95% CI 1.1–8.5, *p* = 0.030, respectively) (Table 4).

**Table 4:** Association between severe cumulative fluid overload and development of expiratory flow limitation according to univariate logistic regression analysis

Variables	Crude odds ratio	95%CI	*p* value
CFO ≥ 10%			
1st to 2nd ICU day of stay	3.9	1.4–10.9	0.011
CFO ≥ 10%			
1st to 3rd ICU day of stay	3.1	1.1–8.5	0.030
AKI in ICU	2.2	0.8–6.0	0.796
AHRF	0.2	0.1–1.2	0.075
ARDS	0.9	0.2–3.6	0.862
Septic shock	2.5	0.9–6.8	0.077

*CFO* cumulative fluid overload, *AKI* acute kidney injury, *ICU* intensive care unit, *AHRF* acute hypoxaemic respiratory failure, *ARDS* acute respiratory distress syndrome

### Outcomes

Overall, patients who had EFL at admission and developed EFL over the first 72 h of ICU stay were ventilated for a longer time (9 [5–15] vs 7 [3–14] days, *p* = 0.043) and had a longer ICU length of stay (14 [10–19] vs 10 [6–17] days, *p* = 0.034) and higher ICU mortality (17/58 (29%) vs 9/63 (14%), *p* = 0.044) compared to those without EFL.

Patients who developed EFL during the first 3 days of ICU stay were not ventilated for a longer time (8 [3–15] vs 7 [3–14] days, *p* = 0.448) and had no longer ICU length of stay (14 [8–18] vs 10 [6–17] days, *p* = 0.344) and higher ICU mortality (5/21 (24%) vs 9/63 (14%), *p* = 0.310) compared to patients who never developed EFL.

## Discussion

The main results of the present study can be summarised as follows: (1) EFL is frequent among ICU patients requiring mechanical ventilation for acute respiratory failure of different origin; (2) patients exhibiting EFL have worse parameters of respiratory mechanics and clinical outcome compared to those who did not; (3) the absence of EFL at ICU admission does not exclude its occurrence during ICU stay since part of the patients (17%) developed EFL after ICU admission; and (4) the development of EFL during the ICU stay was strongly associated with a positive fluid balance.

The presence of EFL was previously detected in 93% of the COPD patients at ICU admission [8], and their pathophysiological pulmonary characteristics explain why they are prone to develop EFL compared to other categories of patients. However, the presence of EFL has been previously demonstrated in other patients so that it could be hypothesised that an unknown amount of ICU patients other than COPD can exhibit EFL. This could have relevant clinical consequences since the presence of EFL has numerous side effects [1], such as the presence of PEEPi [32] that might have detrimental effects on respiratory efficiency and cardiovascular function. Further, the reduction of the expiratory flow and the inability to increase it by the expiratory muscle contraction decrease the efficacy of cough and secretion removal [14] favouring the development of atelectasis, bronchitis and pneumonia. Finally, EFL might imply cyclic opening/closure of the small airways [7, 33] that can lead to hypoxaemia and ventilation/perfusion mismatch.

Interestingly, a large amount of the patients enrolled in the present study (48%) were flow limited within the first 72 h of ICU stay, suggesting that EFL is common in ICU patients. Patients with EFL had higher duration of mechanical ventilation, ICU length of stay and ICU mortality. These outcomes were associated with a more compromised respiratory function since these patients exhibited increased inspiratory and additional resistance and higher PEEPi.

However, our study shows another complementary aspect that deserves clinical attention. We were surprised that 21 patients (17%) became flow limited after ICU admission. While it is easily explainable that obese patients or those with COPD or heart disease can exhibit EFL at ICU admission, it could be less clear why patients might develop EFL during the ICU stay. Interestingly, the main determinant of EFL after ICU admission was a positive fluid balance. Patients who developed EFL during the first 72 h of ICU stay had the higher cumulative fluid overload (Table 4 and Fig. 2); further, a CFO ≥ 10% over the first 2 days of ICU stay was independently associated with EFL (OR 3.7, 95%CI 1.2–11.4, *p* = 0.025).

Hence, fluid therapy can have relevant clinical consequences even at the lung level. Physician should pay particular attention to the amount of fluid administered. Excessive fluids administration can lead to EFL. The latter has been demonstrated to be responsible of damage of small airways that elicit an inflammatory response [22, 23]. It was previously demonstrated that a positive fluid balance can worsen respiratory function, increase the occurrence of pulmonary complication and have an impact on patients’ outcome in patients with acute lung injury and ARDS [34–36].

Detecting and abolishing EFL should be part of the lung-protective strategy. Ventilation at low lung volume leading to EFL could be avoided by the use of PEEP. In patients with flow limited at the ICU admission, the value of PEEP able to avoid EFL was 8 [6–10] cmH_2_O and then statistically decreased to 6 [5–8] at day 3, while in those developing EFL during the ICU stay, this value was 5 [5–6] cmH_2_O at day 1 and 5 [4–7] at day 3. The effects of PEEP on EFL have been previously tested in patients with ARDS. Koutsoukou et al. [37] demonstrated that 10 cmH_2_O of PEEP abolished the presence of EFL during tidal ventilation. The difference between the two studies could be the patients’ population, the severity of the underlying disease and the number of patients enrolled. Koutsoukou et al. [37] studied 13 patients while we enrolled 121 patients with ARF of different aetiologies.

The present study has some limitations: (1) it is a single-centre design with limited sample size. However, this is the first study aiming at identifying possible causes of EFL occurrence in an unselected cohort of ventilated patients; (2) we did not use other techniques such as extra-vascular lung water measurement or lung ultrasound to quantify lung oedema for confirming the association between cumulative fluid overload and EFL occurrence; (3) application of the PEEP test, as it is for all tests evaluating the presence of EFL, carries the need of having the patients for one breath at ZEEP. This could partially derecruit the lung, although the limited time on ZEEP ventilation should minimise the possible effects of the PEEP test on lung function; and (4) we have reported some data on the association between EFL prevalence/development and ICU mortality. However, these data should be regarded only as descriptive since the observational nature of this study and the small sample size do not allow us to make any conclusion. Future larger studies are needed to prove the potential effect of EFL development on the increase of ICU mortality.

## Conclusions

The presence of EFL is common among ICU patients requiring mechanical ventilation for acute respiratory failure of different aetiologies. Interestingly, the major determinant for developing EFL in patients during the first 3 days of their ICU stay is a positive fluid balance. Further studies are needed to assess if a restrictive fluid therapy might be associated with a lower incidence of EFL.

## Supplementary information

**Additional file 1.** Flow chart of the study

**Additional file 2.** Flow-volume loops during positive end-expiratory pressure (PEEP) test

**Additional file 3.** Clinical and Demographic Characteristics of the Patients Enrolled grouped according to the absence / presence of EFL

**Additional file 4.** “PEEP-EFL” values calculated according to the PEEP test in patients with EFL.

**Additional file 5.** Respiratory mechanics characteristic of the patients who developed EFL during the ICU stay.

**Additional file 6.** Cumulative fluid balance and cumulative fluid overload over the first 3 days of ICU stay.

## Data Availability

The datasets used and/or analysed during the current study are available from the corresponding author on reasonable request.

## Abbreviations

AHRF: Acute hypoxaemic respiratory failure
AKI: Acute kidney injury
ARDS: Acute respiratory distress syndrome
BMI: Body mass index
CFB: Cumulative fluid balance
CFO: Cumulative fluid overload
CKD: Chronic kidney disease
CI: Confidence interval
COPD: Chronic obstructive pulmonary disease
Cst,rs: Static compliance of the respiratory system
ΔP: Driving pressure
ΔRrs: Additional resistance of the respiratory system
EFL: Expiratory flow limitation
EPP: Equal pressure point
KDIGO: Kidney Disease Improving Global Outcomes
IBW: Ideal body weight
ICU: Intensive care unit
MRC: Modified Medical Research Council
NYHA: New York Heart Association classification
OR: Odds ratio
OSAS: Obstructive sleep apnoea syndrome
PEEP: Positive end-expiratory pressure
PEEPappl: Positive end-expiratory pressure applied at the ventilator
PEEPi: Intrinsic positive end-expiratory-pressure
P/F: Arterial partial oxygen pressure to fraction of inspired oxygen ratio
Ppeak: Peak inspiratory pressure
Pplat: Plateau pressure
RR: Respiratory rate
Rrs,max: Total resistance of the respiratory system
Rrs,min: Flow resistance of the respiratory system
SAPS: Simplified Acute Physiology Score
SOFA: Sequential Organ Failure Assessment
V_T_: Tidal volume
ZEEP: Zero-PEEP
