# Supplementary material for: Expiratory flow limitation in intensive care: prevalence and risk factors
Source: Crit Care. 2019 Dec 5;23:395. doi: 10.1186/s13054-019-2682-4 (PMC6896682; doi:10.1186/s13054-019-2682-4)
Supplement: Supplementary file 1 — Additional file 1. Flow chart of the study [file 13054_2019_2682_MOESM1_ESM.docx]

**Additional File 1 – Flow chart of the study**
