# Supplementary material for: Expiratory flow limitation in intensive care: prevalence and risk factors
Source: Crit Care. 2019 Dec 5;23:395. doi: 10.1186/s13054-019-2682-4 (PMC6896682; doi:10.1186/s13054-019-2682-4)

**Additional file 2 – Flow-volume loops during positive end-expiratory pressure (PEEP) test.** Flow–volume curves of two representative patients. Panel A: the subtraction of 3 cm H_2_O of PEEP did not increase the expiratory flow and hence the patient was classified as having expiratory flow limitation (EFL). Panel B: the subtraction of 3 cm H_2_O of PEEP increased the expiratory flow and hence the patient was classified as not flow limited.

(ZEEP: PEEP = 0 cm H_2_O)


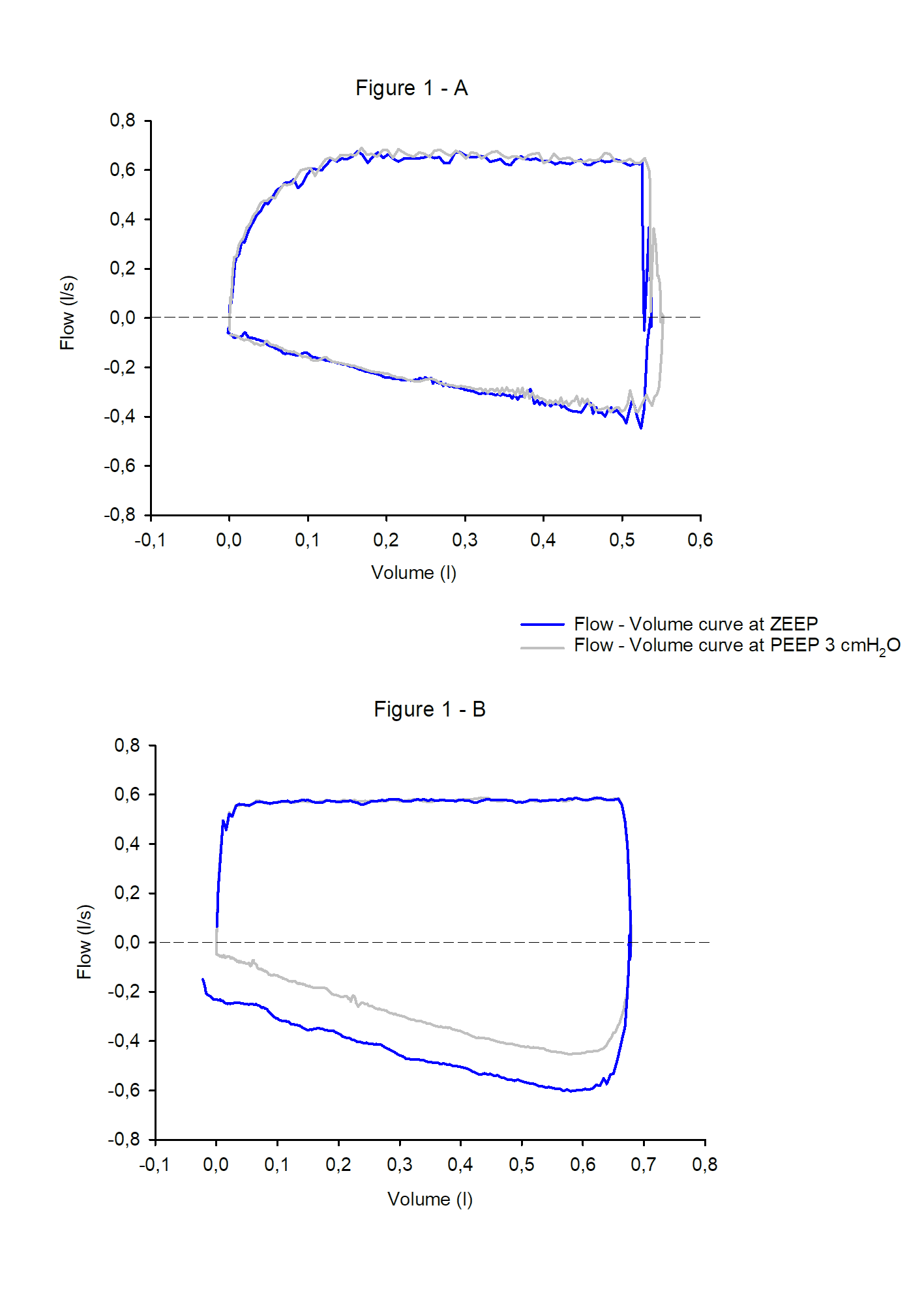

Supplement: Supplementary file 2 — Additional file 2. Flow-volume loops during positive end-expiratory pressure (PEEP) test [file 13054_2019_2682_MOESM2_ESM.docx]
