# Supplementary material for: Expiratory flow limitation in intensive care: prevalence and risk factors
Source: Crit Care. 2019 Dec 5;23:395. doi: 10.1186/s13054-019-2682-4 (PMC6896682; doi:10.1186/s13054-019-2682-4)
Supplement: Supplementary file 3 — Additional file 3. Clinical and Demographic Characteristics of the Patients Enrolled grouped according to the absence / presence of EFL [file 13054_2019_2682_MOESM3_ESM.docx]

**Additional file 3 - Clinical and Demographic Characteristics of the Patients Enrolled grouped according to the absence / presence of EFL**

|  | NO EFL  (n = 63) | EFL  (n = 37) | EFL in  (n = 21) | *p-value* |
| --- | --- | --- | --- | --- |
| Age | 65 ± 15 # | 71 ± 12 | 74 ± 11 | 0.013 |
| Male sex, n (%) | 45 (71) | 21 (57) | 15 (71) | 0.287 |
| BMI (kg/m^2^) | 25.9 ± 4.1 * | 30.7 ± 6.4 # | 25.4 ± 3.6 | < 0.0001 |
| SOFA | 6 [4 – 8] * | 8 [6 – 10] | 8 [4 – 10] | 0.019 |
| SAPSII | 36 [28 – 45] *, # | 42 [35 – 53] | 43 [36 – 58] | 0.003 |
| Smoking history, n (%) |  |  |  | 0.162 |
| Current smoker | 10 (16) | 12 (32) | 7 (33) |  |
| Former smoker | 16 (25) | 8 (22) | 7 (33) |  |
| mMRC *≥ 3* | 4 (6) | 26 (70) | 5 (24) | < 0.0001 |
| NYHA *≥ 2* | 15 (24) | 35 (95) | 12 (57) | < 0.0001 |
| Comorbidities, n (%) |  |  |  |  |
| *Heart diseases* | 25 (40) | 28 (76) | 15 (71) | 0.001 |
| *Hypertension* | 17 (27) | 14 (38) | 11 (52) | 0.095 |
| *Chronic cardiac ischemia* | 16 (25) | 21 (57) | 6 (29) | 0.005 |
| *COPD* | 7 (11) | 19 (51) | 2 (10) | < 0.0001 |
| *OSAS* | 1 (2) | 4 (11) | 2 (10) | 0.117 |
| *CKD* | 6 (10) | 11 (30) | 4 (19) | 0.035 |
| Reason for MV initiation, n (%) |  |  |  |  |
| *AHRF* | 27 (43) | 21 (57) | 4 (19) | 0.021 |
| *Sepsis* | 22 (35) | 11 (30) | 12 (57) | 0.100 |
| *Septic shock* | 13 (21) | 7 (19) | 8 (38) | 0.199 |
| *Hemorragic shock* | 5 (8) | 3 (8) | 3 (14) | 0.660 |
| *Coma* | 9 (14) | 2 (5) | 2 (10) | 0.376 |
| ARDS | 10 (16) | 16 (43) | 3 (14) | 0.004 |
| *Mild* | 1 (10) | 6 (38) | 1 (33) |  |
| *Moderate* | 6 (60) | 6 (38) | 2 (67) |  |
| *Severe* | 3 (30) | 4 (25) | 0 |  |

EFL = expiratory flow limitation; NO EFL: patients never experiencing EFL; EFL: patients with EFL at ICU admission; EFL in: patients becoming flow limited within 3 days after ICU admission; BMI = Body Mass Index; SOFA = Sequential Organ Failure Assessment; SAPS II = Simplified Acute Physiology Score; mMRC = modified Medical Research Council dyspnoea scale; NYHA = New York Heart Association classification; COPD = chronic obstructive pulmonary disease; OSAS = obstructive sleep apnoea syndrome; CKD = chronic kidney disease; ICU = intensive care unti; ARF = acute respiratory failure.

* p < 0.05 compared to With EFL

# p < 0.05 compared to EFL in
