# Supplementary material for: Expiratory flow limitation in intensive care: prevalence and risk factors
Source: Crit Care. 2019 Dec 5;23:395. doi: 10.1186/s13054-019-2682-4 (PMC6896682; doi:10.1186/s13054-019-2682-4)
Supplement: Supplementary file 4 — Additional file 4. “PEEP-EFL” values calculated according to the PEEP test in patients with EFL. [file 13054_2019_2682_MOESM4_ESM.docx]

**Additional file 4 – “PEEP-EFL” values calculated according to the PEEP test in patients with EFL.**

|  | **N. of pts** | **PEEP-EFL day 1** | **N. of pts** | **PEEP-EFL day 2** | **N. of pts** | **PEEP-EFL day 3** | ***p–value*** |
| --- | --- | --- | --- | --- | --- | --- | --- |
| **EFL** | 37 | 8 [6 – 10] | 37 | 7 [6 – 8]* | 37 | 6 [5 – 8]§ | 0.016 |
| **EFLin** | - |  | 12 | 5 [5 – 6] | 21 | 5 [4 – 7] | 0.670 |

EFL = expiratory flow limitation; EFLin: patients who developed EFL after ICU admission; PEEP = positive end expiratory pressure: PEEP-EFL: level of PEEP able to abolish EFL.

* EFL vs EFLin, p = 0.007; § EFL vs EFLin, p = 0.071
