# Supplementary material for: Expiratory flow limitation in intensive care: prevalence and risk factors
Source: Crit Care. 2019 Dec 5;23:395. doi: 10.1186/s13054-019-2682-4 (PMC6896682; doi:10.1186/s13054-019-2682-4)
Supplement: Supplementary file 5 — Additional file 5. Respiratory mechanics characteristic of the patients who developed EFL during the ICU stay. [file 13054_2019_2682_MOESM5_ESM.docx]

**Additional file 5 – Respiratory mechanics characteristic of the patients who developed EFL during the ICU stay.**

|  | **Before EFL**  **(n = 21)** | **EFL**  **(n = 21)** | ***p–value*** |
| --- | --- | --- | --- |
| **Cst,rs**, ml/cmH_2_O | 39 [32 – 56] | 38 [32 – 59] | 0.799 |
| **Rrs,max,** cmH_2_O/l/s | 17 [13 – 21] | 18 [16 – 21] | 0.722 |
| **Rrs,min,** cmH_2_O/l/s | 8 [7 – 13] | 9 [5 – 13] | 0.533 |
| **ΔRrs,** cmH_2_O/l/s | 7 [6 – 10] | 9 [5 – 13] | 0.262 |
| **P/F ratio** | 234 [145 – 289] | 210 [154 – 317] | 0.498 |
| **PEEP_i_,** cmH_2_O | 3 [1 – 5] | 3 [1 – 6] | 0.435 |
| **PEEPappl,** cmH_2_O | 6 [6 – 8] | 8 [6 – 10] | 0.229 |
| **RR,** breaths/min | 15 [15 – 18] | 15 [15 – 18] | 0.443 |
| **V_T_,** ml/kg IBW | 6.4 [5.4 – 7.4] | 6.1 [6.0 – 7.0] | 0.610 |
| **Ppeak,** cmH_2_O | 23 [19 – 26] | 24 [22 – 27] | 0.754 |
| **Pplat,** cmH_2_O | 18 [16 – 20] | 20 [14 – 23] | 0.878 |
| **ΔP,** cmH_2_O | 12 [9 – 13] | 11 [8 – 13] | 0.320 |

EFL = expiratory flow limitation; Cst,rs = static compliance of the respiratory system; Rrs,max = total resistance of the respiratory system; Rrs,min = flow resistance of the respiratory system; ΔRrs = additional resistance of the respiratory system; P/F = arterial partial oxygen pressure to fraction of inspired oxygen ratio; PEEPi = intrinsic positive end expiratory pressure; PEEP appl = positive end expiratory pressure applied at the ventilator; RR = respiratory rate; V_T_ = tidal volume; IBW = ideal body weight; Ppeak: peak inspiratory pressure; Pplat: plateau pressure; **Δ**P: driving pressure.
