# Supplementary material for: Expiratory flow limitation in intensive care: prevalence and risk factors
Source: Crit Care. 2019 Dec 5;23:395. doi: 10.1186/s13054-019-2682-4 (PMC6896682; doi:10.1186/s13054-019-2682-4)
Supplement: Supplementary file 6 — Additional file 6. Cumulative fluid balance and cumulative fluid overload over the first 3 days of ICU stay. [file 13054_2019_2682_MOESM6_ESM.docx]

**Additional file 6 – Cumulative fluid balance and cumulative fluid overload over the first 3 days of ICU stay.**

|  | ***1st ICU day of stay*** | | |  | ***2nd ICU day of stay*** | | |  | ***3rd ICU day of stay*** | | |  |  |
| --- | --- | --- | --- | --- | --- | --- | --- | --- | --- | --- | --- | --- | --- |
|  | ***NoEFL***  *(63)* | ***EFL***  *(37)* | ***EFLin***  *(21)* | ***p^**^*** | ***NoEFL***  *(63)* | ***EFL***  *(37)* | ***EFLin***  *(21)* | ***p^**^*** | ***NoEFL***  *(63)* | ***EFL***  *(37)* | ***EFLin***  *(21)* | ***p^**^*** | ***Trend**** |
| **CFB,** *ml* | 1935  [1000 – 4445] | 1650  [425 – 3197] | 3730  [1917 – 7060] | *0.003* | 4111  [2125 – 6750] | 3255  [1399 –5067] | 6790  [3412 – 9335] | *0.005* | 5193  [2568 – 9155] | 3905  [2002 – 6890] | 7955  [5927 –12350] | *0.002* | *<0.0001* |
| **CFB,** *ml/kg* | 29.7  [13.3 – 59.3] | 23.3  [5.5 – 40.8] | 47.0  [26.6 – 93.0] | *0.002* | 57.0  [31.3 – 92.2] | 39.0  [17.2 –59.8] | 101.2  [53.0 – 129.7] | *0.001* | 65.8  [33.2 –114.4] | 53.8  [26.1 – 86.7] | 103.3  [73.0 – 177.6] | *0.001* | *<0.0001* |
| **CFO,** *%* | 3.0 [1.3 – 5.9] | 2.2 [0.5 – 3.8] | 4.7 [2.7 – 9.3] | *0.001* | 5.7 [3.1 – 9.4] | 3.9 [1.8 – 6.0] | 10.1 [5.3 – 13.0] | *0.001* | 6.8 [3.3 – 12.2] | 5.4 [2.6 – 8.7] | 11.6 [7.3 – 17.8] | *0.001* | *<0.0001* |

EFL = expiratory flow limitation; CFB = cumulative fluid balance; CFO = cumulative fluid overload

*Friedman test was used to test differences within groups among three different timepoints (24h, 48h e 72h from ICU admission).

**Kruskall wallis test and Mann-Whitney test (§) were used to test differences between groups at three different timepoints (24h, 48h e 72h from ICU admission):

§ **Cumulative fluid balance, ml**

1st ICU day of stay: NoEFL vs EFL p = 0.268, NoEFL vs EFL_in_ p = 0.005, EFL vs EFL_in_ p = 0.001.

2nd ICU day of stay: NoEFL vs EFL p = 0.159, NoEFL vs EFL_in_ p = 0.018, EFL vs EFL_in_ p = 0.001.

3rd ICU day of stay: NoEFL vs EFL p = 0.198, NoEFL vs EFL_in_ p = 0.006, EFL vs EFL_in_ < 0.001.

§ **Cumulative fluid balance, ml/kg**

1st ICU day of stay: NoEFL vs EFL p = 0.176, NoEFL vs EFL_in_ p = 0.004, EFL vs EFL_in_ p = 0.001.

2nd ICU day of stay: NoEFL vs EFL p = 0.044, NoEFL vs EFL_in_ p = 0.019, EFL vs EFL_in_ p < 0.001.

3rd ICU day of stay: NoEFL vs EFL p = 0.073, NoEFL vs EFL_in_ p = 0.006, EFL vs EFL_in_ p < 0.001.

§ **CFO, %**

1st ICU day of stay: NoEFL vs EFL p = 0.091, NoEFL vs EFL_in_ p = 0.005, EFL vs EFL_in_ < 0.001.

2nd ICU day of stay: NoEFL vs EFL p = 0.040, NoEFL vs EFL_in_ p = 0.023, EFL vs EFL_in_ < 0.001.

3rd ICU day of stay: NoEFL vs EFL p = 0.065, NoEFL vs EFL_in_ p = 0.006, EFL vs EFL_in_ < 0.001.
